# Diagnosis of Reverse-Connection Defects in High-Voltage Cable Cross-Bonded Grounding System Based on ARO-SVM

**DOI:** 10.3390/s25020590

**Published:** 2025-01-20

**Authors:** Yuhao Ai, Bin Song, Shaocheng Wu, Yongwen Li, Li Lu, Linong Wang

**Affiliations:** 1 Hubei Key Laboratory of Power Equipment & System Security for Integrated Energy, School of Electrical Engineering and Automation, Wuhan University, Wuhan 430072, China; 2023282070150@whu.edu.cn (Y.A.); wushaocheng@whu.edu.cn (S.W.); 2024282070189@whu.edu.cn (Y.L.); luli_ee@whu.edu.cn (L.L.); wangln@whu.edu.cn (L.W.); 2 School of Electrical Engineering and Automation, Wuhan University, Wuhan 430072, China

**Keywords:** high-voltage cable, defect diagnosis, sheath current, ARO

## Abstract

High-voltage (HV) cables are increasingly used in urban power grids, and their safe operation is critical to grid stability. Previous studies have analyzed various defects, including the open circuit in the sheath loop, the flooding in the cross-bonded link box, and the sheath grounding fault. However, there is a paucity of research on the defect of the reverse direction between the inner core and the outer shield of the coaxial cable. Firstly, this paper performed a theoretical analysis of the sheath current in the reversed-connection state and established a simulation model for verification. The outcomes of the simulation demonstrate that there are significant variations in the amplitudes of the sheath current under different reversed-connection conditions. Consequently, a feature vector was devised based on the amplitude of the sheath current. The support vector machine (SVM) was then applied to diagnose the reversed-connection defects in the HV cable cross-bonded grounding system. The artificial rabbits optimization (ARO) algorithm was adopted to optimize the SVM model, attaining an impressively high diagnostic accuracy rate of 99.35%. The effectiveness and feasibility of the proposed algorithm are confirmed through the analysis and validation of the practical example.

## 1. Introduction

The scale of power transmission and distribution grids has been increasing year by year with the development of urbanization. HV cables are widely used due to their advantages, such as meeting the requirements of electric energy transmission and protecting the urban landscape, and their proportion in the power grid is becoming larger and larger [1,2]. Therefore, the safe operation of cables plays an irreplaceable role in ensuring the stability of the power grid [3,4,5].

Power grid workers usually refer to single-core coaxial cables with a rated working voltage of 110 kV and above as HV cables. During operation, the principle of electromagnetic induction states that an induced voltage will appear on the metal sheath of the cable [6]. This induced voltage will change due to the influence of the core current and the cable length. The metal shielding layer of HV cables can reduce the mutual influence between the internal and external electric fields. The grounding methods usually adopted include direct grounding, grounding at both ends, and cross-bonded grounding [7]. The use of cross-bonded grounding can effectively limit the excessive induced voltage and sheath current of HV cables. In the maintenance and inspection of the HV cable cross-bonded grounding system, the sheath current is one of the important parameters reflecting the cable state. Based on the changes in the sheath current, cable fault states can be effectively diagnosed [8,9,10].

Damage to the sheath, incorrect wiring in link boxes, the puncture of epoxy prefabricated parts, and the reversal of the direction of the core and outer shielding layer of coaxial cables are all reasons that can cause changes in the sheath current of the HV cable cross-bonded grounding system, affecting the normal operation of the cables [11,12]. In identifying abnormal sheath currents in HV cable cross-bonded grounding systems, personnel are required to undertake a range of diagnostic procedures to ascertain the nature and location of cable faults. These include partial discharge monitoring [13], insulation resistance monitoring [14], grounding current monitoring [15], etc. This process can significantly impact the stability of the power system. Consequently, the expeditious diagnosis of cable fault types and the swift identification of fault locations are of paramount importance [16]. Scholars both domestically and internationally have conducted research and analysis in various directions regarding the cross-bonded grounding systems of HV cables. Reference [17] proposed a methodology for analyzing the cable sheath current, utilizing the separation of the cable sheath current and the resistive current from the leakage current to determine the presence of a cable fault. This approach was substantiated through the utilization of both simulation and experimental methods. However, it should be noted that the method is constrained in its capacity to analyze a wide range of cable faults, encompassing only cable insulation faults, cable sheath loop faults, and cable joint puncture faults. Reference [18] constructed a feature vector based on the amplitude and phase angle of the sheath current in the grounding boxes at both ends, and employed the long short-term memory (LSTM) algorithm to perform the intelligent classification of faults in the cable cross-bonded grounding system. However, in practical engineering, the phase angle of the sheath current was rarely used to assess cable faults, and the proposed method required verification. Reference [19] proposed a fault location method based on the electrical quantities at both cable ends. A substantial body of simulation results demonstrated the capability of this method to locate the fault starting point accurately. However, the data analysis of the proposed method was relatively complex. Reference [20] introduced the defect of the coaxial cable core and shielding direction being reversed, and analyzed its circulating current characteristics. Although it achieved the classification and generalization of common reversal defects, the leakage current was overlooked, only the induced current was considered, and it did not employ simulation for validation.

Current online detection methods for cable faults typically involve the construction of feature vectors based on a substantial number of feature quantities to facilitate the identification of faults. However, these methods are deficient in their coverage of fault types, particularly in the context of coaxial cable core and outer shielding layer reversals, a phenomenon that has received scant attention in the extant literature. When the connection direction between the cable core and the outer shielding layer is reversed, the circulating current in the sheath will increase significantly, leading to operational failures. This will further endanger the stability of the power system, potentially causing power outages and economic losses. Furthermore, the utilization of intelligent algorithms, such as machine learning and deep learning, in the field of cable fault diagnosis for cross-bonded grounding systems is also limited. Among them, due to its excellent classification performance, strong nonlinear processing ability, and high anti-interference capacity, the SVM has been widely applied in the field of fault diagnosis [21,22]. However, the selection of hyperparameters in the SVM directly affects the results of the fault diagnosis.

Therefore, this paper proposed a diagnosis method for reverse-connection defects in the cross-bonded grounding system of HV cables based on an optimized SVM algorithm. The main contributions of this paper were as follows:

1. The reverse-connection defects between the core and the outer shielding layer of coaxial cables were systematically integrated. The theoretical situations of sheath currents under different reverse-connection states were analyzed, providing theoretical guidance for grid workers to diagnose reverse-connection defects in the cable cross-bonded grounding system.
2. A simulation model of the cable cross-bonded grounding system was built using the Power System Computer-Aided Design (PSCAD) simulation software, version v4.6.2. The theoretical situations of sheath currents under different reverse-connection states were verified. It was found that the amplitude of the sheath current varied significantly under different operating conditions, providing a basis for the subsequent construction of feature vectors.
3. A feature vector based on the amplitude of the sheath current was constructed. The ARO algorithm optimized the SVM model and compared it with the particle swarm optimization (PSO) and sparrow search algorithm (SSA). This improved the accuracy and efficiency of multi-classification fault diagnosis, and the accuracy rate was increased by 5.84% compared with the basic model.

## 2. Analysis of Sheath Currents Under Normal Operation

**Figure 1:**
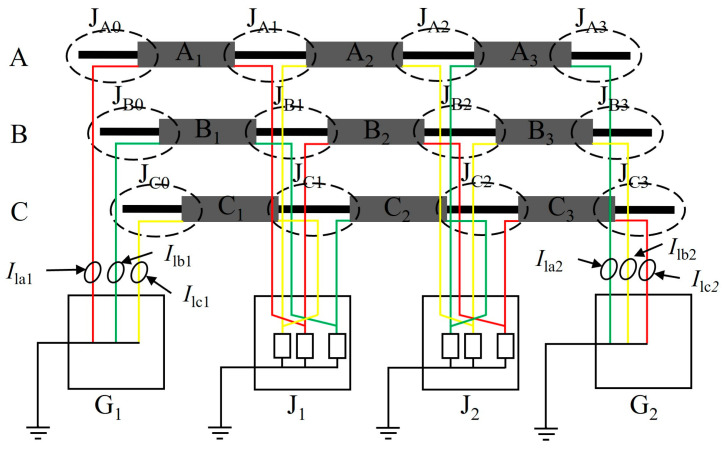
Model of cross-bonded grounding system for HV cables.

In HV cable lines with a length greater than 1000 m, the metal sheath grounding system of HV cables generally employed a cross-bonded method to reduce the induced voltage and sheath current. The configuration of the HV cable cross-bonded grounding system was illustrated in Figure 1.

Figure 1 showed a cross-bonded main section, and a typical HV cable line generally consists of several such sections. Each main section contained two direct grounding boxes at the ends (G_1_ and G_2_), two cross-bonded grounding boxes (J_1_ and J_2_), nine minor metallic sheath sections of approximately equal length (A_1_, A_2_,…, C_3_), and twelve cable joints (J_A0_, J_A1_,…, J_C3_). The sheath of the HV cable was transposed utilizing cross-bonded boxes, forming three sheath return loops: A_1_-B_2_-C_3_, B_1_-C_2_-A_3_, and C_1_-A_2_-B_3_. The cable sheath currents, designated *I*_la1_, *I*_lb1_, *I*_lc1_, *I*_la2_, *I*_lb2_, and *I*_lc2_, could be divided into leakage currents [23] and sheath-induced currents. These sheath currents flowed directly to the earth through G_1_ and G_2_, completing the closed circuit.

### 2.1. Leakage Current

**Figure 2:**
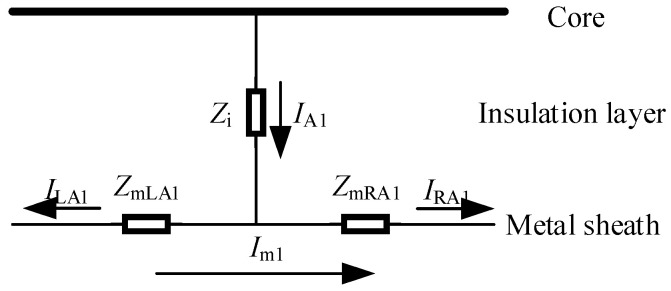
Schematic diagram of the flow direction of the leakage current of the cable in minor section A_1_.

The flow direction of the leakage current of the cable was from the cable core through the insulating layer to the metal sheath. Taking minor section A_1_ as an example for the introduction, its flow direction schematic diagram was shown in Figure 2.

In Figure 2, *I*_A1_ represented the leakage current of the cable, while *I*_LA1_ and *I*_RA1_ were the components of the leakage current flowing to the left and right in the metal sheath, respectively. Their equivalent relationship could be seen in Equation (1). *Z*_i_ was the equivalent impedance of the insulating layer, and *Z*_mLA1_ and *Z*_mRA1_ were the equivalent impedances of the left and right sides of the cable metal sheath, respectively.

(1) $$I_{A1}=I_{\mathrm{LA}1}+I_{\mathrm{RA}1}$$

(2) $$Z_{\mathrm{mA}1}=Z_{\mathrm{mLA}1}+Z_{\mathrm{mRA}1}$$

where *Z*_mA1_ was the equivalent impedance of the minor section A1 of the metal sheath.

Since the capacitive current accounted for a relatively large proportion of the leakage current, the leakage current was approximated as the capacitive current:

(3) $$I_{A1}\approx I_{\mathrm{CA}1}=jwCU_{A}l_{A1}$$

where *I*_CA1_ represented the capacitive current, *U*_A_ was the voltage of the cable core, *l*_A1_ was the length of the minor section A_1_ of the metal sheath, and C was the capacitance of the cable with its value obtainable from Equation (4). Since the voltage of the HV cable cross-bonded grounding system and the impedance of the cable insulating layer would not change in a short period, the cable leakage current could be considered approximately constant.

(4) $$C=\frac{\varepsilon_{r}\times 5.56\times 10^{-11}}{\ln\left(\frac{D_{C}\;+\;2\delta }{D_{C}}\right)}$$

where *ε*_r_ was the relative dielectric constant, *D*_C_ was the diameter of the cable core, and *σ* was the thickness of the insulator. The relative dielectric constant of the cross-linked polyethylene was 2.3.

The leakage current components in the sheath loop could be calculated according to the current distribution rule. Take section A_1_ as an example—*I*_LA1_ and *I*_RA1_ could be obtained as follows:

(5) $$\left\{\begin{array}{l} I_{\mathrm{LA}1}=\frac{Z_{\mathrm{mRA}1}+Z_{\mathrm{mB}2}+Z_{\mathrm{mC}3}+R_{g}}{Z_{\mathrm{mA}1}+Z_{\mathrm{mB}2}+Z_{\mathrm{mC}3}+R_{e}+R_{g}}I_{A1}\\ I_{\mathrm{RA}1}=\frac{Z_{\mathrm{mLA}1}+R_{e}}{Z_{\mathrm{mA}1}+Z_{\mathrm{mB}2}+Z_{\mathrm{mC}3}+R_{e}+R_{g}}I_{A1} \end{array}\right.$$

where *Z*_mA1_, *Z*_mB2,_ and *Z*_mC3_ were the equivalent impedances of the three minor sections A_1_, B_2_, and C_3_ of the metal sheath, respectively, and *R*_e_ and *R*_g_ were the grounding resistances at both ends of the cross-bonded loop.

### 2.2. Sheath-Induced Current

**Figure 3:**
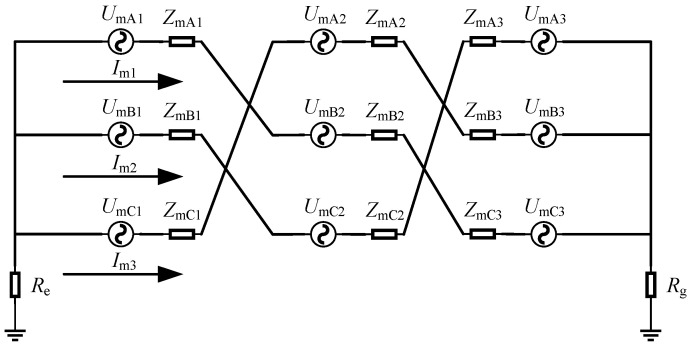
Equivalent induced circuit of the metal sheath of the cross-bonded cable.

The current generated in the sheath loop based on the principle of electromagnetic induction was called the sheath-induced current. The equivalent induced circuit of the metal sheath of the cross-bonded cable was shown in Figure 3.

In the figure, *U*_mAn_, *U*_mBn_, and *U*_mCn_ (n = 1, 2, 3) were the induced voltages of the metal sheaths of each section of the cable. *Z*_mAn_, *Z*_mBn_, and *Z*_mCn_ were the equivalent impedances of the metal sheaths of each section of the cable, respectively, and *I*_m1_, *I*_m2_, and *I*_m3_ were the induced circulating currents of the three sheath loops whose values can be calculated by Equation (6).

(6) $$\left\{\begin{array}{l} I_{m1}=\frac{U_{\mathrm{mA}1}+U_{\mathrm{mB}2}+U_{\mathrm{mC}3}}{Z_{\mathrm{mA}1}+Z_{\mathrm{mB}2}+Z_{\mathrm{mC}3}+R_{e}+R_{g}}\\ I_{m2}=\frac{U_{\mathrm{mB}1}+U_{\mathrm{mC}2}+U_{\mathrm{mA}3}}{Z_{\mathrm{mB}1}+Z_{\mathrm{mC}2}+Z_{\mathrm{mA}3}+R_{e}+R_{g}}\\ I_{m3}=\frac{U_{\mathrm{mC}1}+U_{\mathrm{mA}2}+U_{\mathrm{mB}3}}{Z_{\mathrm{mC}1}+Z_{\mathrm{mA}2}+Z_{\mathrm{mB}3}+R_{e}+R_{g}} \end{array}\right.$$

Taking minor section A_1_ as an example to calculate its induced voltage, the length of the HV cable, the method of installation, and the core current could all have an effect on the induced voltage of the sheath. However, since the induced current was much smaller than the core current, the effect of the sheath-induced current on the induced voltage was not considered. The calculation of its induced voltage was as follows:

(7) $$U_{\mathrm{mA}1}=-jw\left(I_{A}L_{\mathrm{AA}}+I_{B}L_{\mathrm{AB}}+I_{C}L_{\mathrm{AC}}\right)l_{A1}$$

where *I*_A_, *I*_B_, and *I*_C_ were the cable core currents of phases A, B, and C, respectively. *L*_AA_ was the mutual inductance coefficient of the core of phase A to the metal sheath of phase A, *M*_AB_ was the mutual inductance coefficient of the core of phase B to the metal sheath of phase A, and *M*_AC_ was the mutual inductance coefficient of the core of phase C to the metal sheath of phase A.

### 2.3. Current in the Grounding Boxes

The sheath current in the grounding boxes at both ends was the vector sum of the leakage current and the sheath induced current. This paper stipulated that the positive direction of the current flow is to the right. The change of the sheath current in the grounding box could reflect the operation status of the sheath loop. According to the above analysis, the sheath current in the grounding box was as shown in Equation (8). When ideal conditions such as the same cable laying method, equal lengths of cross-bonded sections, and balanced core load currents were met, the induced voltages on each section of the metal sheath generally satisfied the following relationships—*U*_mA1_ = *U*_mA2_ = *U*_mA3_, *U*_mB1_ = *U*_mB2_ = *U*_mB3_, *U*_mC1_ = *U*_mC2_ = *U*_mC3_—and the magnitudes of *U*_mAn_, *U*_mBn_, and *U*_mCn_ were equal. The amplitude when the three voltages were equal was designated *U*_m_. The phase of *U*_mAn_ preceded the phase of *U*_mBn_ by 120°, and the phase of *U*_mBn_ succeeded the phase of *U*_mCn_ by 120°. It was evident that the magnitude and phase relationships of the voltages of each phase core were similar. Furthermore, the equivalent impedances of each section of the metal sheath were approximately equal. We denoted this by *Z*_m_. It was evident that *I*_m1_, *I*_m2_, and *I*_m3_ were close to 0 when the cable functioned within normal parameters.

Consequently, when the cable functioned within normal parameters, the sheath current within the grounding enclosure was predominantly influenced by the leakage current.

(8) $$\left\{\begin{array}{l} I_{\mathrm{la}1}=-\left(I_{\mathrm{LA}1}+I_{\mathrm{LB}2}+I_{\mathrm{LC}3}\right)+I_{m1}\\ I_{\mathrm{lb}1}=-\left(I_{\mathrm{LB}1}+I_{\mathrm{LC}2}+I_{\mathrm{LA}3}\right)+I_{m2}\\ I_{\mathrm{lc}1}=-\left(I_{\mathrm{LC}1}+I_{\mathrm{LA}2}+I_{\mathrm{LB}3}\right)+I_{m3}\\ I_{\mathrm{la}2}=\left(I_{\mathrm{RB}1}+I_{\mathrm{RC}2}+I_{\mathrm{RA}3}\right)+I_{m2}\\ I_{\mathrm{lb}2}=\left(I_{\mathrm{RC}1}+I_{\mathrm{RA}2}+I_{\mathrm{RB}3}\right)+I_{m3}\\ I_{\mathrm{lc}2}=\left(I_{\mathrm{RA}1}+I_{\mathrm{RB}2}+I_{\mathrm{RC}3}\right)+I_{m1} \end{array}\right.$$

where *I*_la1_ and *I*_lc2_ were the currents at the beginning and end of the sheath loop A_1_-B_2_-C_3_, *I*_lb1_ and *I*_la2_ were the currents at the beginning and end of the sheath loop B_1_-C_2_-A_3_, and *I*_lc1_ and *I*_lb2_ were the currents at the beginning and end of the sheath loop C_1_-A_2_-B_3_.

## 3. Analysis of Sheath Currents for Reverse-Connection Defects

**Figure 4:**
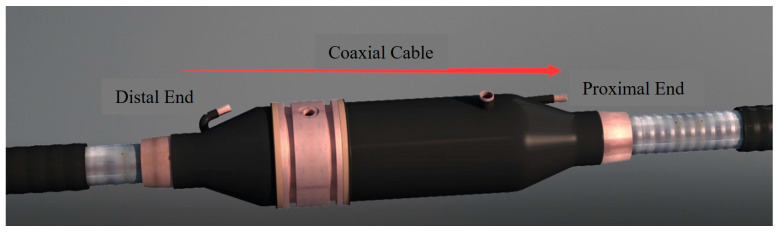
Schematic diagram of coaxial cable joint.

In a normal cross-bonded grounding system, the inner core and outer shielding layer of the grounded coaxial cable are connected to the aluminum sheath at the far and near ends, respectively. Typically, we designate the aluminum sheath along the direction of the coaxial cable as the proximal end, and the opposite end as the distal end, as illustrated in Figure 4. If there is a reverse-connection defect between the inner core and outer shielding layer of the coaxial cable, the induced voltage of the cable metal sheath cannot be canceled out. This results in an increase in the sheath current and a reduction in the service life of the insulation. The following analysis will examine the sheath current of the reverse-connection defect, focusing primarily on one reverse connection and two reverse connections. The specific reverse-connection defect states are shown in Table 1. Since the probability of three or more simultaneous reverse connections is extremely low, this paper does not consider it.

### 3.1. One Reversal

**Figure 5:**
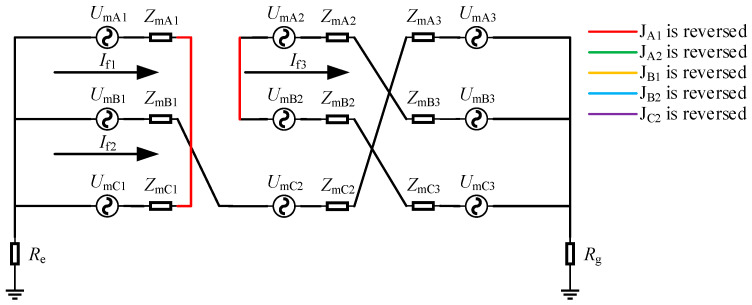
Equivalent circuit diagram when J_A1_ is reversed.

There was a total of six types of single-reversal defects where the inner core and outer shielding layer of the coaxial cable were reversed: J_A1_, J_B1_, J_C1_, J_A2_, J_B2_, and J_C2_ were reversed. Taking the reverse-connection defect of the cable joint J_A1_ as an example for analysis, its equivalent circuit diagram was shown in Figure 5.

When the J_A1_ had a reverse-connection defect, the sheath circuit changed. There were three circuits in total. Loop 1 is A_1_-C_1_, loop 2 is B_1_-C_2_-A_3_, and loop 3 is A_2_-B_3_-C_3_-B_2_. The induced current in the sheath would also change accordingly, as shown in Equation (9). The induced sheath current of loop 2 was consistent with that during normal operation and its magnitude was close to 0. Since the impedance of loop 3 was 4*Z*_m_ and the impedance of loop 1 was 2*Z*_m_, the induced current of loop 1 was twice that of loop 3.

(9) $$\left\{\begin{array}{l} I_{f1}=\frac{U_{\mathrm{mA}1}-U_{\mathrm{mC}1}}{Z_{\mathrm{mA}1}+Z_{\mathrm{mC}1}}\\ I_{f2}=I_{m2}\\ I_{f3}=\frac{U_{\mathrm{mA}2}+U_{\mathrm{mB}3}-U_{\mathrm{mC}3}-U_{\mathrm{mB}2}}{Z_{\mathrm{mA}2}+Z_{\mathrm{mB}3}+Z_{\mathrm{mC}3}+Z_{\mathrm{mB}2}} \end{array}\right.$$

where *I*_f1_, *I*_f2_, and *I*_f3_ were the induced currents in the sheath circuits under the current defect condition.

It could be seen from Figure 5 that the impedances of loops 1 and 3 had changed, so the distribution of the leakage current in each section of these two circuits would also change accordingly. Taking minor section A1 as an example, the change in its leakage current component was shown in Equation (10) (the leakage current components of each small section in other reverse-connection defects could be written using the current distribution rule, so no further analysis was carried out). Since loop 2 had not changed, its sheath current could still be obtained by Equation (8). Based on the above equations, the current in the grounding box could be obtained as shown in Equation (11). Since the leakage current was smaller than the induced current, the sheath current magnitude could be judged by the change in induced current. Among them, *I*_la1_ and *I*_lc1_ were at the two ends of the same sheath loop, so their magnitudes and phases were approximately the same. Similarly, it could be obtained that *I*_lb2_ and *I*_lc2_ were also approximately equal, and *I*_lb1_ and *I*_la2_ were also approximately equal. Obviously, at this time, *I*_lb1_ and *I*_la2_ were close to the current values during normal operation and were much smaller than the other four values, while *I*_la1_ and *I*_lc1_ would be greater than *I*_lb2_ and *I*_lc2_.

(10) $$\left\{\begin{array}{l} I_{\mathrm{LA}1}=\frac{Z_{\mathrm{mRA}1}+Z_{\mathrm{mC}1}}{Z_{\mathrm{mA}1}+Z_{\mathrm{mC}1}}I_{A1}\\ I_{\mathrm{RA}1}=\frac{Z_{\mathrm{mLA}1}}{Z_{\mathrm{mA}1}+Z_{\mathrm{mC}1}}I_{A1} \end{array}\right.$$

(11) $$\left\{\begin{array}{l} I_{\mathrm{la}1}=-\left(I_{\mathrm{LA}1}+I_{\mathrm{RC}1}\right)+I_{f1}\\ I_{\mathrm{lb}1}=-\left(I_{\mathrm{LB}1}+I_{\mathrm{LC}2}+I_{\mathrm{LA}3}\right)+I_{f2}\\ I_{\mathrm{lc}1}=-\left(I_{\mathrm{LC}1}+I_{\mathrm{RA}1}\right)-I_{f1}\\ I_{\mathrm{la}2}=\left(I_{\mathrm{RB}1}+I_{\mathrm{RC}2}+I_{\mathrm{RA}3}\right)+I_{f2}\\ I_{\mathrm{lb}2}=\left(I_{\mathrm{RA}2}+I_{\mathrm{RB}3}+I_{\mathrm{LB}2}+I_{\mathrm{LC}3}\right)+I_{f3}\\ I_{\mathrm{lc}2}=\left(I_{\mathrm{LA}2}+I_{\mathrm{LB}3}+I_{\mathrm{RB}2}+I_{\mathrm{RC}3}\right)-I_{f3} \end{array}\right.$$

### 3.2. Two Reversals

There were three categories of defects in which the inner core and outer shielding layer of the coaxial cable were reversed at two positions: reversed connections within the same cross-bonded link box, reversed connections of the same phase, and reversed connections at joints across different phases and cross-bonded link boxes.

#### 3.2.1. Reversed Connections Within the Same Cross-Bonded Link Box

**Figure 6:**
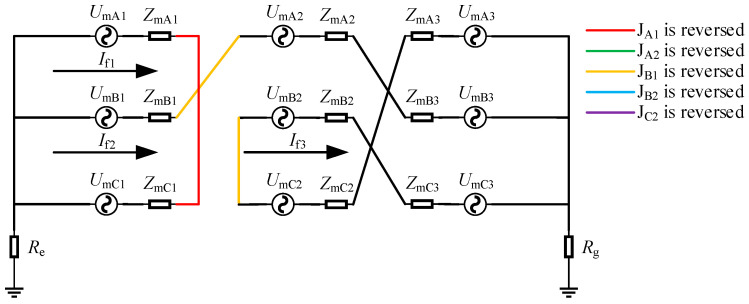
Equivalent circuit diagram when J_A1_ and J_B1_ are reversed.

There was a total of six cases where two reversed connections of the inner core and outer shielding layer of the coaxial cable were located within the same cross-bonded link box: J_A1_ and J_B1_ were reversed simultaneously, J_A1_ and J_C1_ were reversed simultaneously, J_B1_ and J_C1_ were reversed simultaneously, J_A2_ and J_B2_ were reversed simultaneously, J_A2_ and J_C2_ were reversed simultaneously, and J_B2_ and J_C2_ were reversed simultaneously. Taking the case where J_A1_ and J_B1_ were reversed simultaneously as an example for analysis, the equivalent circuit diagram of this situation was shown in Figure 6.

When J_A1_ and J_B1_ were reversed simultaneously, there were three sheath loops: loop 1 was A_1_-C_1_, loop 2 was B_1_-A_2_-B_3_, and loop 3 was B_2_-C_3_-A_3_-C_2_. Since the sheath circuits were inconsistent with those in normal operation, both the induced current in the sheath and the leakage current would change. It could be obtained from Figure 6 that the induced current in the sheath of the faulty circuit was as follows:

(12) $$\left\{\begin{array}{l} I_{f1}=\frac{U_{\mathrm{mA}1}-U_{\mathrm{mC}1}}{Z_{\mathrm{mA}1}+Z_{\mathrm{mC}1}}\\ I_{f2}=\frac{U_{\mathrm{mB}1}+U_{\mathrm{mA}2}+U_{\mathrm{mB}3}}{Z_{\mathrm{mB}1}+Z_{\mathrm{mA}2}+Z_{\mathrm{mB}3}+R_{e}+R_{g}}\\ I_{f3}=\frac{U_{\mathrm{mB}2}+U_{\mathrm{mC}3}-U_{\mathrm{mA}3}-U_{\mathrm{mC}2}}{Z_{\mathrm{mB}2}+Z_{\mathrm{mC}3}+Z_{\mathrm{mA}3}+Z_{\mathrm{mC}2}} \end{array}\right.$$

There was a grounding resistance in loop 2, and the grounding resistance was much larger than the equivalent impedance of the sheath. Therefore, *I*_f2_ was the smallest. The impedance of loop 1 was twice that of loop 3, so the magnitude of *I*_f1_ was twice that of *I*_f3_, and there was a 120° phase difference between them.

According to the superposition theorem, the current in the grounding box was as follows:

(13) $$\left\{\begin{array}{l} I_{\mathrm{la}1}=-\left(I_{\mathrm{LA}1}+I_{\mathrm{RC}1}\right)+I_{f1}\\ I_{\mathrm{lb}1}=-\left(I_{\mathrm{LB}1}+I_{\mathrm{LA}2}+I_{\mathrm{LB}3}\right)+I_{f2}\\ I_{\mathrm{lc}1}=-\left(I_{\mathrm{LC}1}+I_{\mathrm{RA}1}\right)-I_{f1}\\ I_{\mathrm{la}2}=\left(I_{\mathrm{LB}2}+I_{\mathrm{LC}3}+I_{\mathrm{RA}3}+I_{\mathrm{RC}2}\right)-I_{f3}\\ I_{\mathrm{lb}2}=\left(I_{\mathrm{RB}1}+I_{\mathrm{RA}2}+I_{\mathrm{RB}3}\right)+I_{f2}\\ I_{\mathrm{lc}2}=\left(I_{\mathrm{RB}2}+I_{\mathrm{RC}3}+I_{\mathrm{LA}3}+I_{\mathrm{LC}2}\right)+I_{f3} \end{array}\right.,$$

As shown in Figure 6, *I*_la1_ and *I*_lc1_ were located at the two ends of loop 1, so they were approximately equal. Similarly, it could be obtained that *I*_lb1_ and *I*_lb2_ were also approximately equal, and *I*_la2_ and *I*_lc2_ were also approximately equal. Judging from the magnitude relationship of the induced currents: *I*_lb1_ and *I*_lb2_ were greater than the current values during normal operation but smaller than the other four sheath currents. *I*_la1_ and *I*_lc1_ were the largest among the sheath currents at this time and were approximately equal to the corresponding current values when J_A1_ was reversed. Due to the influence of leakage current, the phase difference between *I*_la1_ and *I*_lc2_, as well as between *I*_la2_ and *I*_lc1_, was not 120°.

#### 3.2.2. Reversed Connections of the Same Phase

**Figure 7:**
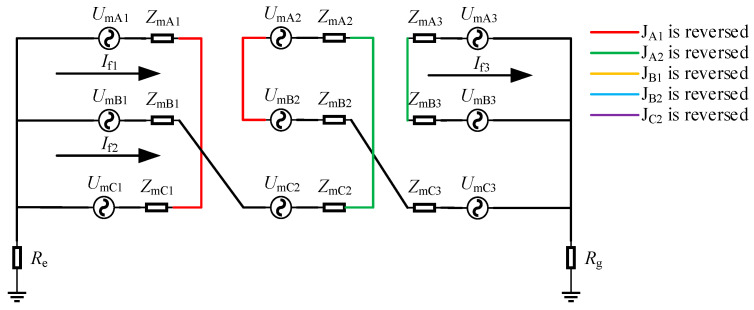
Equivalent circuit diagram when J_A1_ and J_A2_ are reversed.

There was a total of three cases where the two reversed connections of the inner core and outer shielding layer of the coaxial cable were of the same phase: J_A1_ and J_A2_ were reversed, J_B1_ and J_B2_ were reversed, and J_C1_ and J_C2_ were reversed. Taking the case where J_A1_ and J_A2_ were reversed as an example for introduction, the equivalent circuit diagram of this situation was shown in Figure 7.

When J_A1_ and J_A2_ were reversed simultaneously, the sheath circuit was different from that in normal operation. Loop 1 was A_1_-C_1_, loop 2 was B_1_-C_2_-A_2_-B_2_-C_3_, and loop 3 was A_3_-B_3_. The induced current in the sheath of the corresponding circuit and the leakage current of each minor section of the metal sheath also changed accordingly. It could be obtained from Figure 7 that the induced current in the sheath of the faulty circuit was as follows:

(14) $$\left\{\begin{array}{l} I_{f1}=\frac{U_{\mathrm{mA}1}-U_{\mathrm{mC}1}}{Z_{\mathrm{mA}1}+Z_{\mathrm{mC}1}}\\ I_{f2}=\frac{U_{\mathrm{mB}1}+U_{\mathrm{mC}2}-U_{\mathrm{mA}2}+U_{\mathrm{mB}2}+U_{\mathrm{mC}3}}{Z_{\mathrm{mB}1}+Z_{\mathrm{mC}2}+Z_{\mathrm{mA}2}+Z_{\mathrm{mB}2}+Z_{\mathrm{mC}3}+R_{e}+R_{g}}\\ I_{f3}=\frac{U_{\mathrm{mA}3}-U_{\mathrm{mB}3}}{Z_{\mathrm{mA}3}+Z_{\mathrm{mB}3}} \end{array}\right.,$$

As could be seen from Equation (14), the impedances of loops 1–3 were 2*Z*_m_, 5*Z*_m_ + *R*_e_ + *R*_g_, and 2*Z*_m_, respectively. Since the grounding resistance was much larger than the sheath impedance, the current of loop 2 was much smaller than that of the other loops. However, at this time, *I*_f2_ was slightly larger than the induced current value of loop 2 when J_A1_ and J_B1_ were reversed, while the magnitudes of *I*_f1_ and *I*_f3_ were equal.

According to the superposition theorem, the current in the grounding box was as follows:

(15) $$\left\{\begin{array}{l} I_{\mathrm{la}1}=-\left(I_{\mathrm{LA}1}+I_{\mathrm{RC}1}\right)+I_{f1}\\ I_{\mathrm{lb}1}=-\left(I_{\mathrm{LB}1}+I_{\mathrm{LC}2}+I_{\mathrm{RA}2}+I_{\mathrm{LB}2}+I_{\mathrm{LC}3}\right)+I_{f2}\\ I_{\mathrm{lc}1}=-\left(I_{\mathrm{LC}1}+I_{\mathrm{RA}1}\right)-I_{f1}\\ I_{\mathrm{la}2}=\left(I_{\mathrm{RA}3}+I_{\mathrm{LB}2}\right)+I_{f3}\\ I_{\mathrm{lb}2}=\left(I_{\mathrm{LA}3}+I_{\mathrm{RB}3}\right)-I_{f3}\\ I_{\mathrm{lc}2}=\left(I_{\mathrm{RB}1}+I_{\mathrm{RC}2}+I_{\mathrm{LA}2}+I_{\mathrm{RB}2}+I_{\mathrm{RC}3}\right)+I_{f2} \end{array}\right.$$

Since the topology of loop 1 was consistent with that of loop 1 when J_A1_ and J_B1_ were reversed, and the topology of loop 3 was similar to it as well, the sheath currents were approximately equal. However, loop 2 had undergone significant changes and contained five minor sections of the metal sheath. Judging from the relationship of the induced sheath currents, the magnitudes of *I*_la1_, *I*_lc1_, *I*_la2_, and *I*_lb2_ were approximately equal, while the current values of *I*_lb1_ and *I*_lc2_ were the smallest.

#### 3.2.3. Reversed Connections at Joints Across Different Phases and Cross-Bonded Grounding Boxes

There was a total of six cases where the two reversed connections of the inner core and outer shielding layer of the coaxial cable were located at joints across different phases and cross-bonded grounding boxes: J_A1_ and J_B2_ were reversed simultaneously, J_A1_ and J_C2_ were reversed simultaneously, J_B1_ and J_A2_ were reversed simultaneously, J_B1_ and J_C2_ were reversed simultaneously, J_C1_ and J_A2_ were reversed simultaneously, and J_C1_ and J_B2_ were reversed simultaneously. According to the reversed topology, it could be further divided into two cases. One case was that J_A1_ and J_B2_ are reversed simultaneously, J_B1_ and J_C2_ were reversed simultaneously, and J_C1_ and J_A2_ were reversed simultaneously. The other case was that J_B1_ and J_A2_ were reversed simultaneously, J_C1_ and J_B2_ were reversed simultaneously, and J_A1_ and J_C2_ were reversed simultaneously.

**Figure 8:**
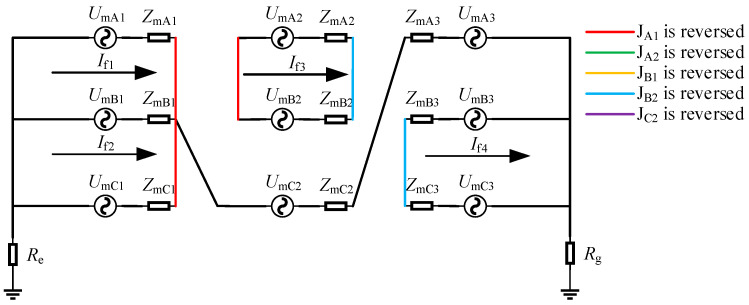
Equivalent circuit diagram when J_A1_ and J_B2_ are reversed.

Take the simultaneous reversal of J_A1_ and J_B2_ as an example for the first case. The equivalent circuit diagram under this circumstance was shown in Figure 8.

When J_A1_ and J_B2_ were reversed simultaneously, there are four sheath loops: loop 1 was A_1_-C_1_, loop 2 was B_1_-C_2_-A_3_, loop 3 was A_2_-B_2_, and loop 4 was B_3_-C_3_. Since loop 2 operated as in normal conditions, the induced current and leakage current in loop 2 remained unchanged, while those in other sheath circuits varied. It could be obtained from Figure 8 that the induced current in the sheath of the faulty circuit was as follows:

(16) $$\left\{\begin{array}{l} I_{f1}=\frac{U_{\mathrm{mA}1}-U_{\mathrm{mC}1}}{Z_{\mathrm{mA}1}+Z_{\mathrm{mC}1}}\\ I_{f2}=\frac{U_{\mathrm{mB}1}+U_{\mathrm{mC}2}+U_{\mathrm{mA}3}}{Z_{\mathrm{mB}1}+Z_{\mathrm{mC}2}+Z_{\mathrm{mA}3}+R_{e}+R_{g}}\\ I_{f3}=\frac{U_{\mathrm{mA}2}-U_{\mathrm{mB}2}}{Z_{\mathrm{mA}2}+Z_{\mathrm{mB}2}}\\ I_{f4}=\frac{U_{\mathrm{mB}3}-U_{\mathrm{mC}3}}{Z_{\mathrm{mB}3}+Z_{\mathrm{mC}3}} \end{array}\right.$$

Loop 2 eliminated the induced voltage through a three-phase superposition. The voltages and impedances of the remaining loops were consistent. Therefore, the induced current of loop 2 was zero, and the magnitudes of the induced currents of the remaining loops were equal.

According to the superposition theorem, the current in the grounding box was as follows:

(17) $$\left\{\begin{array}{l} I_{\mathrm{la}1}=-\left(I_{\mathrm{LA}1}+I_{\mathrm{RC}1}\right)+I_{f1}\\ I_{\mathrm{lb}1}=-\left(I_{\mathrm{LB}1}+I_{\mathrm{LC}2}+I_{\mathrm{LA}3}\right)+I_{f2}\\ I_{\mathrm{lc}1}=-\left(I_{\mathrm{LC}1}+I_{\mathrm{RA}1}\right)-I_{f1}\\ I_{\mathrm{la}2}=\left(I_{\mathrm{RB}1}+I_{\mathrm{RC}2}+I_{\mathrm{RA}3}\right)+I_{f2}\\ I_{\mathrm{lb}2}=\left(I_{\mathrm{RB}3}+I_{\mathrm{LC}3}\right)+I_{f4}\\ I_{\mathrm{lc}2}=\left(I_{\mathrm{LB}3}+I_{\mathrm{RC}3}\right)-I_{f4} \end{array}\right.$$

**Figure 9:**
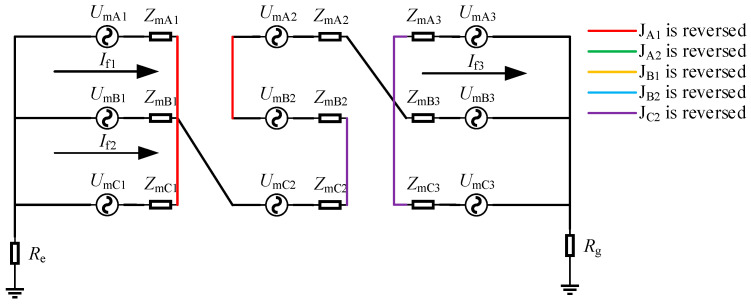
Equivalent circuit diagram when J_A1_ and J_C2_ are reversed.

As could be seen from Figure 9, *I*_la1_ and *I*_lc1_ were located at the two ends of loop 1, so they were approximately equal. Similarly, it could be obtained that I_lb1_ and I_la2_ were also approximately equal, and *I*_lb2_ and *I*_lc2_ were also approximately equal. Loop 3 did not pass through the grounding box, so the current of this loop was not analyzed. Since the induced current value of loop 2 was zero, its sheath current was only the leakage current. It could be known that *I*_lb1_ and *I*_la2_ were close to zero. The magnitudes of the induced currents of loop 1 and loop 4 were equal, so the magnitudes of *I*_la1_, *I*_lc1_, *I*_lb2_, and *I*_lc2_ were also approximately equal.

Another case was analyzed by taking the simultaneous reversal of J_A1_ and J_C2_ as an example. The equivalent circuit diagram under this circumstance was shown in Figure 9.

When J_A1_ and J_C2_ were reversed simultaneously, there were three sheath loops in total. Loop 1 was A_1_-C_1_, loop 2 was B_1_-C_2_-B_2_-A_2_-B_3_, and loop 3 was A_3_-C_2_. Loop 2 contained five minor sections of the metal sheath, and both the beginning and the end of loop 2 were of the B phase. The topologies of loop 1 and loop 3 were consistent, both containing the metal sheaths of phases A and C. As could be obtained from Figure 9, the induced sheath currents of the loops were as follows:

(18) $$\left\{\begin{array}{l} I_{f1}=\frac{U_{\mathrm{mA}1}-U_{\mathrm{mC}1}}{Z_{\mathrm{mA}1}+Z_{\mathrm{mC}1}}=\frac{\sqrt{3}U_{m}\times e^{-j30^{\circ }}}{2Z_{m}}\\ I_{f2}=\frac{U_{\mathrm{mB}1}+U_{\mathrm{mC}2}-U_{\mathrm{mB}2}+U_{\mathrm{mA}2}+U_{\mathrm{mB}3}}{Z_{\mathrm{mB}1}+Z_{\mathrm{mC}2}+Z_{\mathrm{mB}2}+Z_{\mathrm{mA}2}+Z_{\mathrm{mB}3}+R_{e}+R_{g}}=0\\ I_{f3}=\frac{U_{\mathrm{mA}3}-U_{\mathrm{mC}3}}{Z_{\mathrm{mA}3}+Z_{\mathrm{mC}3}}=\frac{\sqrt{3}U_{m}\times e^{-j30^{\circ }}}{2Z_{m}} \end{array}\right.$$

As could be known from the above equation, loop 2 eliminated the induced voltage through voltage superposition, thus resulting in its induced current being zero. The induced currents of loop 1 and loop 3 were equal.

(19) $$\left\{\begin{array}{l} I_{\mathrm{la}1}=-\left(I_{\mathrm{LA}1}+I_{\mathrm{RC}1}\right)+I_{f1}\\ I_{\mathrm{lb}1}=-\left(I_{\mathrm{LB}1}+I_{\mathrm{LC}2}+I_{\mathrm{RB}2}+I_{\mathrm{LA}2}+I_{\mathrm{LB}3}\right)+I_{f2}\\ I_{\mathrm{lc}1}=-\left(I_{\mathrm{LC}1}+I_{\mathrm{RA}1}\right)-I_{f1}\\ I_{\mathrm{la}2}=\left(I_{\mathrm{RA}3}+I_{\mathrm{LC}3}\right)+I_{f3}\\ I_{\mathrm{lb}2}=\left(I_{\mathrm{RB}1}+I_{\mathrm{RC}2}+I_{\mathrm{LB}2}+I_{\mathrm{RA}2}+I_{\mathrm{RB}3}\right)+I_{f2}\\ I_{\mathrm{lc}2}=\left(I_{\mathrm{LA}3}+I_{\mathrm{RC}3}\right)-I_{f3} \end{array}\right.$$

As could be seen from Figure 9 and Equation (19), *I*_la1_ and *I*_lc1_ were located at the two ends of loop 1, while *I*_la2_ and *I*_lc2_ were at the two ends of loop 3. The currents at the corresponding beginning and end of the two loops were basically equal. Since the induced current of loop 2 was zero, its sheath current, that is, leakage current, was close to zero and nearly identical to the current magnitude during normal operation.

Although these two reversed-connection cases both occurred in different phases and different cross-bonded grounding boxes, one had four loops while the other had three loops. In both reversed-connection cases, the sheath currents of two loops were basically equal, and the circulating current of the other loop was normal. However, the loop topologies of the two were not completely consistent.

### 3.3. Selection of Feature Quantities

Different reverse-connection defects in the cross-bonded grounding system significantly altered the metal sheath circuit compared to normal operation, thereby affecting the sheath current in the grounding boxes at both ends. Therefore, we could use the sheath current as a feature to judge the operating state of the HV cable. The sheath current included two signals: amplitude and phase. However, in engineering practice, the measurement of the amplitude signal was relatively simple, and the data processing was more straightforward. In contrast, the phase signal was susceptible to electromagnetic interference, and its measurement was more difficult. Moreover, at present, the staff mainly rely on the amplitude signal of the sheath current to determine whether a fault occurs in the cable grounding system. Considering these practical factors, the current phase signal was excluded, and the feature quantities were constructed based solely on the amplitude signal to reflect the sheath circuit’s operating state.

As could be known from the above analysis, during normal operation, the sheath currents *I*_la1_ and *I*_lc2_ jointly reflected the operating state of loop A_1_-B_2_-C_3_, *I*_lb1_ and *I*_la2_ jointly reflected the operating state of loop B_1_-C_2_-A_3_, and *I*_lc1_ and *I*_lb2_ jointly reflected the operating state of loop C_1_-A_2_-B_3_. During the reversed operation, the sheath loops changed, and *I*_la1_, *I*_lc2_, *I*_lb1,_ *I*_la2_, *I*_lc1_, and *I*_lb2_ might not necessarily reflect the operating state of the same loop anymore. Therefore, feature quantities could be constructed based on the amplitudes of the above six currents. To reduce the influence of the cable operating voltage and parameters, the cable ratios were selected as the feature quantities, as shown in Equation (20).

Since the sheath currents *I*_la1_ and *I*_lc2_ jointly reflected the operating state of the loop A_1_-B_2_-C_3_, *I*_lb1_ and *I*_la2_ jointly reflected the operating state of the loop B_1_-C_2_-A_3_, and *I*_lc1_ and *I*_lb2_ jointly reflected the operating state of the loop C_1_-A_2_-B_3_; the feature quantity could be constructed based on the amplitudes of the above six currents, as shown in Equation (20).

(20) $$\left\{\begin{array}{l} t_{1}=\left|\frac{I_{\mathrm{lc}2}-I_{\mathrm{la}1}}{\max\left(I_{\mathrm{la}1},I_{\mathrm{lc}2}\right)}\right|,\;t_{2}=\left|\frac{I_{\mathrm{la}2}-I_{\mathrm{lb}1}}{\max\left(I_{\mathrm{la}2},I_{\mathrm{lb}1}\right)}\right|,\;t_{3}=\left|\frac{I_{\mathrm{lb}2}-I_{\mathrm{lc}1}}{\max\left(I_{\mathrm{lc}1},I_{\mathrm{lb}2}\right)}\right|\\ t_{4}=\frac{I_{\mathrm{la}1}}{I_{\mathrm{lb}1}},\;t_{5}=\frac{I_{\mathrm{lb}1}}{I_{\mathrm{lc}1}},\;t_{6}=\frac{I_{\mathrm{la}1}}{I_{\mathrm{lc}1}} \end{array}\right.$$

where *t*_1_, *t*_2_, and *t*_3_ were the absolute values of the ratio of the amplitude difference of the same sheath circuit to the maximum amplitude of the circuit, which could reflect the operating state of the same metal sheath circuit. *t*_4_, *t*_5_, and *t*_6_ were the amplitude ratios of the sheath current in the grounding box at the front end, which could reflect the operating state of different metal sheath circuits. According to the changes in the above six feature quantities, the type and location of the cable reverse-connection defect could be determined.

Thus, the feature quantity matrix used in this paper to reflect the operating state of the HV cable was as follows:

(21) $$\left[t_{1},t_{2},t_{3},t_{4},t_{5},t_{6}\right]$$

## 4. Simulation Analysis

**Figure 10:**
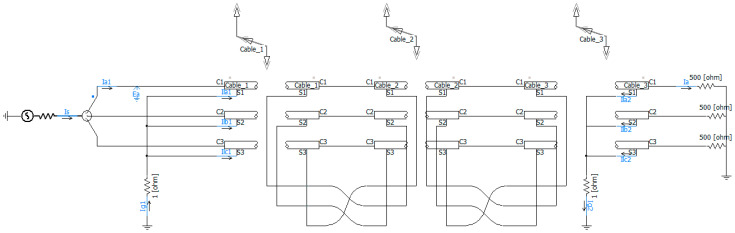
Simulation model.

In this paper, the cable of type YJLW03-64/110-1×800 was chosen for constructing the simulation model. The cables were arranged horizontally, with a burial depth of 1 m and a spacing of 0.27 m, spanning a total length of 1500 m. The three interconnected segments of the cross-bonded system each had a length of 500 m. The specific parameters are shown in Table 2 [24]. In the PSCAD v4.6.2 software, the cable line was modeled using a frequency-dependent model. The voltage level was set at 110 kV, and a three-phase balanced load was configured as a resistive load with a resistance value of 500 Ω. Meanwhile, the grounding resistance of the sheath circuit was 1 Ω. The simulation model built using PSCAD v4.6.2 software was shown in Figure 10.

### 4.1. Simulation of Cable in Normal Operation

**Figure 11:**
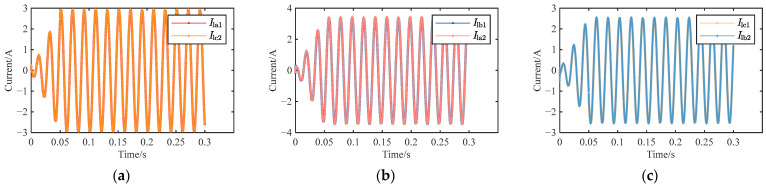
Waveform diagram of sheath current when the cable is in normal operation: (**a**) *I*_la1_ and *I*_lc2_; (**b**) *I*_lb1_ and *I*_la2_; and (**c**) *I*_lc1_ and *I*_lb2_.

According to the ammeter in the simulation model, the conductor current of the cable was 126.875 A. The simulation time was set to 0.3 s. When the cable was in normal operation, the waveforms of the sheath current in the grounding boxes at both ends were shown in Figure 11.

It could be seen from Figure 11 that, when the cable was in normal operation, the sheath currents in the grounding boxes at both ends of each sheath circuit were almost the same. The slight differences in the currents of different sheath circuits were due to the electromagnetic induction phenomenon between the conductor and the sheath. According to the oscilloscope in the simulation model, the sheath currents in the grounding boxes at both ends of different sheath circuits were measured as follows: *I*_la1_ = 2.0883∠152.3° A, *I*_lc2_ = 2.0832∠152.1° A, *I*_lb1_ = 2.433∠35.68° A, *I*_la2_ = 2.437∠35.55° A, *I*_lc1_ = 1.803∠ − 61.86° A, and *I*_lb2_ = 1.805∠ − 61.77° A. The corresponding feature quantity matrix was [0.00244, 0.00164, 0.0011, 0.858, 1.349, 1.158].

### 4.2. Simulation of One-Reversal Operation

**Figure 12:**
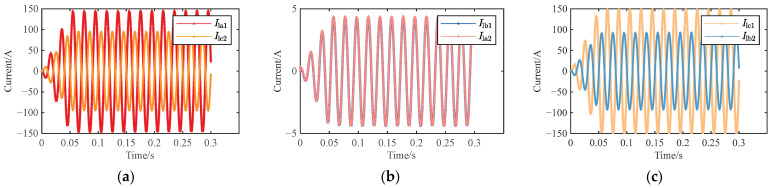
Waveform diagram of sheath current when J_A1_ is reversed: (**a**) *I*_la1_ and *I*_lc2_; (**b**) *I*_lb1_ and *I*_la2_; and (**c**) *I*_lc1_ and *I*_lb2_.

Taking the reverse connection of the inner core and outer shielding layer of the coaxial cable at cable joint J_A1_ as an example for simulation analysis, when the J_A1_ had a reverse-connection defect, the waveforms of the sheath current in the grounding boxes were shown in Figure 12.

Figure 12 showed that, when J_A1_ had a reverse-connection defect, the sheath currents in the grounding boxes at both ends of each sheath circuit changed significantly. However, the amplitudes and phases of *I*_lb1_ and *I*_la2_ were still approximately the same, because the sheath loop 2 was the same as that in normal operation. According to the oscilloscope in the simulation model, the sheath currents in the grounding boxes were measured as follows: *I*_la1_ = 103.581∠81.75° A, *I*_lc2_ = 67.219∠ − 94.67° A, *I*_lb1_ = 3.10845∠49.91° A, *I*_la2_ = 3.11119∠49.8° A, *I*_lc1_ = 105.347∠ − 98.63° A, and *I*_lb2_ = 65.512∠86.04° A. The corresponding feature quantity matrix was [0.351, 0.00088, 0.3781, 33.322, 0.0295, 0.9832].

Based on the above current values, the magnitudes of *I*_la1_ and *I*_lc1_ were nearly identical, with a phase difference of approximately 180°. The magnitudes of *I*_lb1_ and *I*_la2_ were basically the same as those during normal operation, and their phases were also basically equal. Since loop 3 contained four small sections of the metal sheath and loop 2 only contained two, the magnitudes of *I*_lb2_ and *I*_lc2_ were smaller compared to *I*_la1_ and *I*_lc1_.

### 4.3. Simulation of Two-Reversal Operation

**Figure 13:**
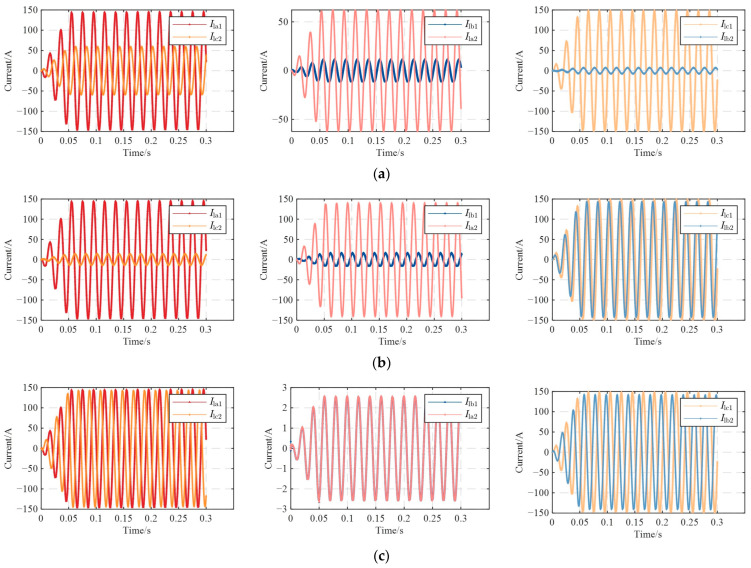

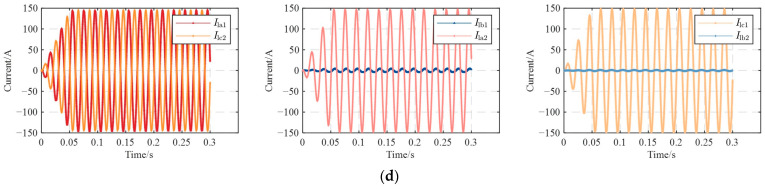
Waveform diagram of sheath current when the cable is in two-reversal operation: (**a**) J_A1_ and J_B1_ are reversed; (**b**) J_A1_ and J_A2_ are reversed; (**c**) J_A1_ and J_B2_ are reversed; and (**d**) J_A1_ and J_C2_ are reversed.

Taking the reverse connection of intermediate joints J_A1_ and J_B1_ as an example when the same cross-bonded grounding box was reversed, and taking the reverse connection of J_A1_ and J_A2_ as an example for the same-phase reverse connection, for the simulation of the reverse connection in different phases and different cross-bonded grounding boxes, the reverse connections of J_A1_ and J_B2_, as well as J_A1_ and J_C2_, were taken as examples. The waveforms of the sheath currents in the grounding box when these two joints were reversed were shown in Figure 13.

When J_A1_ and J_B1_ had a reverse connection, the sheath currents in the direct grounding boxes were measured as follows: *I*_la1_ = 103.583∠81.75° A, *I*_lc2_ = 42.16∠ − 50.69° A, *I*_lb1_ = 8.178∠72.15° A, *I*_la2_ = 44.0269∠128.9° A, *I*_lc1_ = 105.348∠ − 98.64° A, and *I*_lb2_ = 5.14868∠60.85° A. The corresponding feature quantity matrix was [0.59298, 0.8143, 0.9511, 12.666, 0.07763, 0.98325].

When J_A1_ and J_A2_ had a reverse connection, the sheath currents in the grounding boxes were measured as follows: *I*_la1_ = 103.575∠81.75° A and *I*_lc2_ = 9.84574∠ − 28.24° A, *I*_lb1_ = 11.5434∠ − 23.82° A and *I*_la2_ = 99.4441∠132.8° A, *I*_lc1_ = 105.343∠ − 98.64° A, and *I*_lb2_ = 101.32∠ − 47.48° A. The corresponding feature quantity matrix was [0.90494, 0.88392, 0.03838, 8.97266, 0.10958, 0.98322].

When J_A1_ and J_B2_ had reverse-connection defects, the sheath currents in the grounding boxes were measured as follows: *I*_la1_ = 103.532∠81.71° A, *I*_lc2_ = 102.003∠ − 143.2° A, *I*_lb1_ = 1.82066∠10.29° A, *I*_la2_ = 1.82713∠10.18° A, *I*_lc1_ = 105.298∠ − 98.67° A, and *I*_lb2_ = 100.494∠37.42° A. The corresponding feature quantity matrix was [0.014768, 0.003541, 0.045623, 56.8651, 0.017291, 0.98323].

When J_A1_ and J_C2_ had reverse-connection defects, the sheath currents in the grounding boxes were measured as follows: *I*_la1_ = 103.581∠81.76° A, *I*_lc2_ = 102.923∠ − 100.8° A, *I*_lb1_ = 3.06403∠49.64° A, *I*_la2_ = 104.717∠78.83° A, *I*_lc1_ = 105.346∠ − 98.63° A, and *I*_lb2_ = 0.95605∠ − 84.73° A. The corresponding feature quantity matrix was [0.006353, 0.97074, 0.9909, 33.8055, 0.02909, 0.98325].

When J_A1_ and J_B1_ were reversely connected, the loop 2 was B_1_-A_2_-B_3_. Its induced voltage could not cancel each other out, so its amplitude was larger than that of the current during normal operation. Based on the measured current values, the amplitude of loop 2 was clearly larger than that during normal operation. Except for the reverse connection of J_A1_ and J_C2_, loop 1 of the other three reverse-connection cases was the same as that of loop 1 when J_A1_ was reversed. It could be seen from the measured current values that the current of this loop was basically equal to that when J_A1_ was reversed. Since the loop topology in some reverse-connection cases was similar to that of loop 1, its current amplitude was also close to it. The amplitudes measured in loop 2 when J_A1_ and J_B2_ were reversely connected and when J_A1_ and J_C2_ were reversely connected were both close to those during normal operation.

### 4.4. Dataset Construction

Based on the above-built HV cable simulation model, simulations of different defect types were carried out. Under constant cable structure parameters, sheath current data were obtained by varying the cable length and load resistance. The feature quantity matrices reflecting the operating state of the HV cable were constructed according to Equations (20) and (21). The length of each section of the HV cable using the cross-bonded grounding method was generally 500 m. Therefore, the value range of each cable section’s length was set from 400 m to 600 m, and then increased by 50 m successively, resulting in five selection methods. In actual power systems, the load resistance was determined by the equipment used by users. Hence, the value range of the load resistance was set from 500 Ω to 800 Ω, increasing by 50 Ω successively, which leads to seven selection methods. There were 35 parameter combinations for each defect location, and there were 21 defect locations in total. The dataset had 770 groups in total.

The selection of cable length and load resistance was based on the actual operation of HV cables. By designing a reasonable parameter range, both the diversity and representativeness of the simulation dataset were ensured, providing sufficient data support for subsequent defect diagnosis.

## 5. Reverse Defect Diagnosis Model

### 5.1. SVM Model

The SVM is a machine-learning algorithm used for regression and classification [25]. Its core idea is to find an optimal hyperplane to distinguish between different categories of data. By maximizing the margin, the robustness of the algorithm is improved, and it has significant advantages in dealing with problems such as small samples, nonlinearity, and overfitting.

SVM processes nonlinearly separable data through kernel functions. Common kernel functions include the linear kernel function, RBF kernel function, sigmoid kernel function, etc. Due to the excellent generalization ability and wide application of the radial basis function (RBF) kernel function, this paper selects the RBF kernel function. At this time, the accuracy of the SVM model is greatly affected by the penalty factor C and the kernel function parameter g. Adjusting C affects the model’s generalization ability and training speed, while g determines the kernel function’s shape and complexity, influencing data mapping in high-dimensional space. To give the SVM model a better classification ability, appropriate parameters need to be selected to achieve the best effect.

### 5.2. Artificial Rabbits Optimization Algorithm

**Figure 14:**
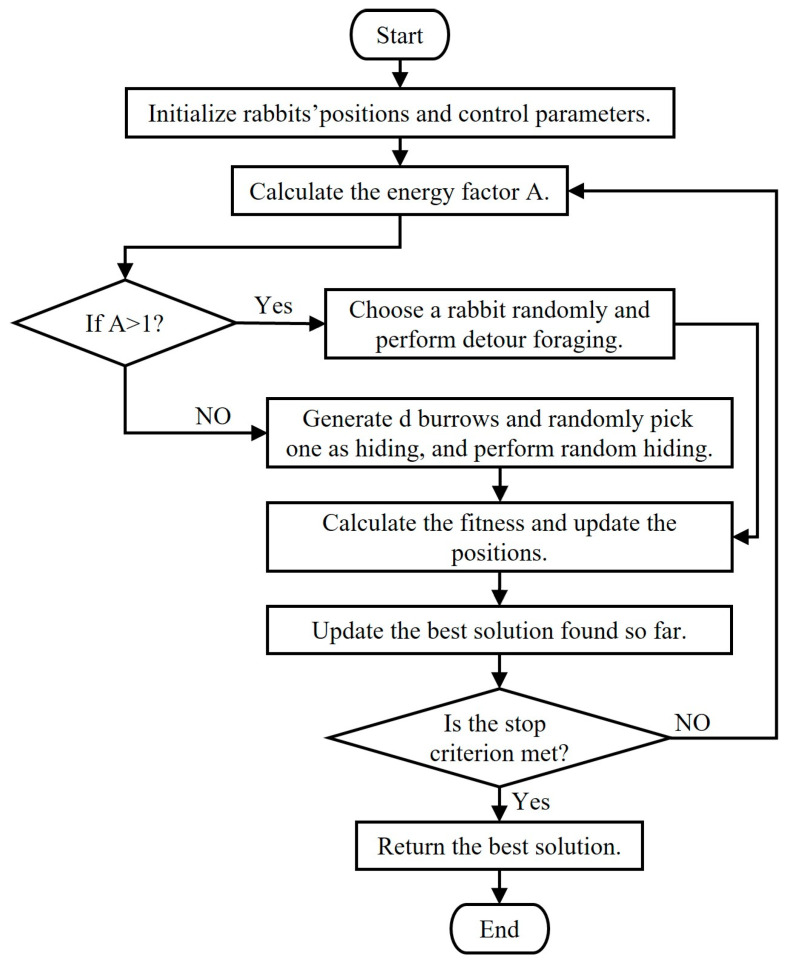
Flowchart of ARO algorithm [26].

The artificial rabbits optimization algorithm was proposed by Liying Wang et al. in 2022 [26]. This algorithm originates from the survival strategies of rabbits in nature. These strategies mainly consist of detouring foraging and random hiding, which are exploration and exploitation, respectively. The switch between them is based on the energy contraction mechanism. To avoid predators detecting their burrow, rabbits forage randomly in areas distant from their burrow during the foraging process. This behavior greatly improves the exploration efficiency and global search ability of the algorithm. At each iteration of the ARO algorithm, each rabbit digs d burrows along each dimension of the search space and randomly selects one in which to hide, promoting search diversity through the random hiding strategy. In the initial stage of iteration, the rabbits in the population tend to detour foraging due to their relatively high energy. As the energy decreases, they will execute the random hiding strategy. In the ARO algorithm, when the energy factor A > 1, the rabbit is in the exploration stage; otherwise, it is in the exploitation stage. After implementing either the detouring foraging or the random hiding strategy, the position of the rabbit is called the candidate position. The actual position of the rabbit will change according to the fitness value between the candidate position and the current position. If the candidate position has better fitness, the rabbit will move to that position; otherwise, it will stay in its current position. The flowchart of the ARO algorithm is shown in Figure 14.

### 5.3. Defect Diagnosis Model Based on ARO-SVM

This paper used the ARO algorithm to optimize the SVM model. The specific diagnosis steps are as follows:

1. The acquisition and preprocessing of sheath current amplitude information. Simulate various defects using PSCAD v4.6.2 software, collect the sheath current amplitude data from the grounding boxes at both ends of the HV cable, and construct feature vectors based on Equations (20) and (21). The formed dataset is then randomly divided into a training set and a test set at a ratio of 4:1.
2. Model training. Firstly, the parameters of the ARO algorithm are initialized. Selecting an appropriate initial population size and maximum number of iterations is crucial to balancing the search accuracy and computational efficiency. The population size was set to 30, and the maximum number of iterations (t_max_) was set to 50. The population size and iteration count should be adjusted based on whether the fitness function converges when the maximum iteration is reached. Select the population strategy based on the energy factor value and calculate the defect diagnosis accuracy after each parameter update. Update the optimal accuracy and parameters until the stopping condition is met and the maximum iterations are reached to obtain the optimal SVM model. Defect identification. Input the test set into the SVM model obtained in the previous step for defect identification.

**Figure 15:**
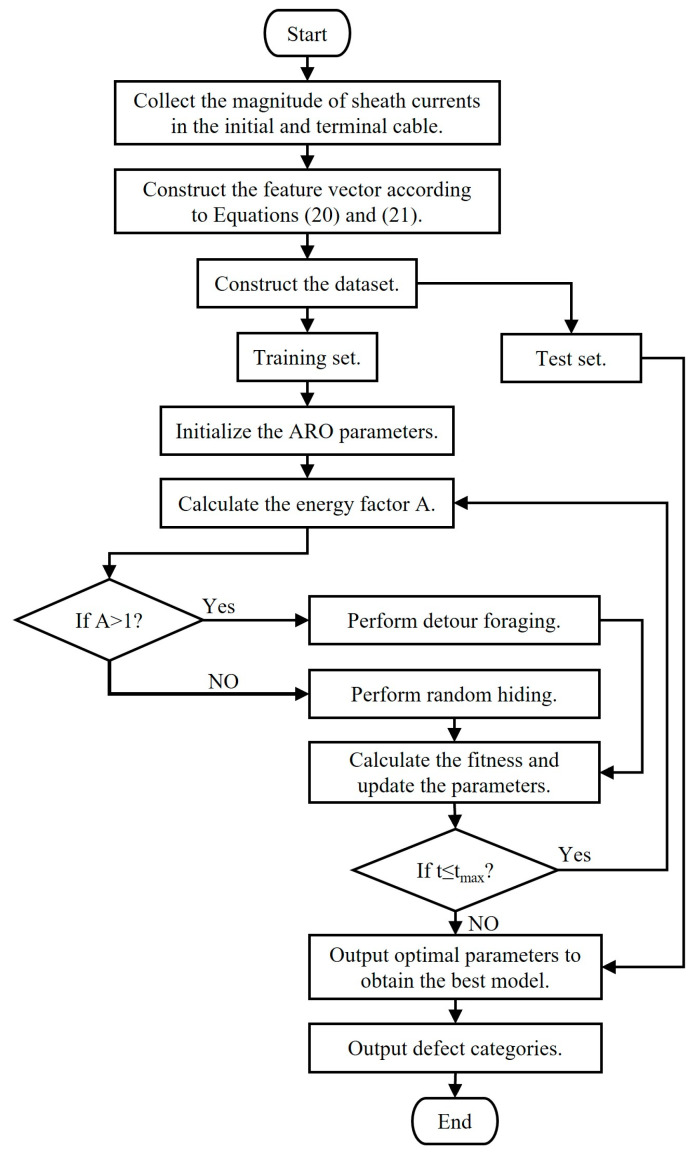
Flowchart of the defect diagnosis model based on ARO-SVM.

The flowchart of the defect diagnosis model based on ARO-SVM was shown in Figure 15.

The optimization effect of the ARO-SVM model could be verified by comparing with the decision tree (DT), extreme gradient boosting (XGBoost), back propagation (BP), SVM, PSO-SVM, and SSA-SVM models through control experiments [27,28,29,30,31]. By inputting the same training set and test set, the defect diagnosis accuracy of each model could be obtained as shown in Table 3.

As shown in Table 3, the defect diagnosis accuracies of the DT, XGBoost, BP, SVM, ARO-DT, ARO-XGBoost, ARO-BP, PSO-SVM, SSA-SVM, and ARO-SVM models were 92.86%, 98.05%, 90.26%, 93.51%, 97.40%, 98.70%, 91.56%, 98.05%, 95.45%, and 99.35%, respectively. The accuracy of the ARO-SVM model was increased by 6.49%, 1.3%, 9.09%, 5.84%, 1.95%, 0.65%, 7.79%, 1.3%, and 3.9% compared with those of the DT, XGBoost, BP, SVM, ARO-DT, ARO-XGBoost, ARO-BP, PSO-SVM, and SSA-SVM models, respectively. Since the ARO-SVM model exhibited the highest accuracy, this paper selected the ARO algorithm to optimize the SVM model. Compared with the SVM model, the ARO-SVM model could accurately identify the defects with state numbers 3, 4, and 6, and the accuracy of the defect with state number 2 was also 90.91%. Therefore, the algorithm proposed in this paper can accurately identify the reverse-connection defects in the HV cable cross-bonded grounding system.

### 5.4. Influence of Noise Interference

In actual operation, noise interference distorted the sheath current waveform of the HV cable cross-bonded grounding system, affecting measurement accuracy. To test the model’s robustness against noise, noise with SNRs of 20, 30, and 40 dB was added to the simulated sheath current waveform. The smaller the SNR, the stronger the noise signal. The feature vectors obtained after adding noise interference were input into the model, and various types of reverse-connection defects were tested. The specific test results were shown in Table 4.

Table 4 showed that, under noise interference with different SNRs, the model accurately identified defect types and demonstrated strong resistance to noise.

### 5.5. Case Verification

In this paper, a 220 kV HV cable line was taken as an example for analysis [32]. The total length of this cable line was 6.77 km, and it adopted the cross-bonded grounding method, including 2 GIS terminals and 11 intermediate joints. After a certain grounding circulating current measurement, the operators found that the data between box No. 9 and the GIS terminal were abnormal. The measured values of the sheath current were shown in Table 5.

**Figure 16:**
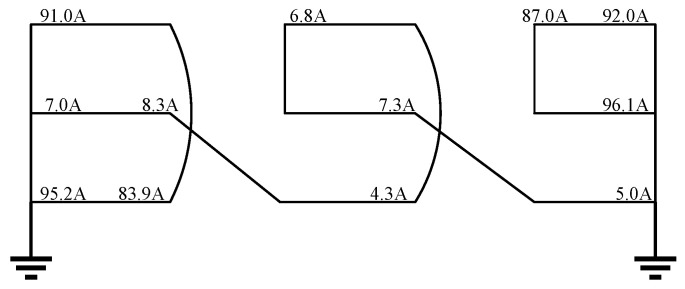
Actual wiring method.

According to the measured values of the sheath current, the actual wiring method causing the defect in the grounding system was shown in Figure 16. It could be clearly seen that the defect was the simultaneous reverse connection of the inner core and outer shielding layer at intermediate joints J_A1_ and J_A2_. This state corresponded to the fault numbered 13 as defined in Table 1 of this paper. Calculate the sheath current feature matrix according to Equations (20) and (21) as [0.094506, 0.92391, 0.009365, 13, 0.07353, 0.95588]. Input it into the ARO-SVM model, and the diagnosis result was 13, which was the same as the actual state number. Therefore, the proposed model effectively diagnoses reverse-connection defects in HV cables and is unaffected by the operating voltage and cable structure parameters.

Existing detection methods [33] can only determine whether a defect has occurred in the HV cable cross-bonded grounding system, but cannot accurately identify the type of defect. In contrast, the algorithm proposed in this paper not only accurately identifies reverse-connection defects in the grounding system but also roughly locates the defect position in the cable, significantly reducing the maintenance time for technicians.

### 5.6. Discussion on Practical Deployment and Challenges

According to the above analysis, the ARO-SVM-based cable reverse-connection defect diagnosis model effectively identifies defects in the sheath grounding system. However, in practical applications, we still need to consider the possible challenges that this model may face.

To integrate the proposed method with the existing HV cable cross-bonded grounding system, current transformers and data acquisition modules must be installed in the grounding boxes at both ends for online sheath current monitoring. Additionally, as the cable system expands, we must further optimize the ARO-SVM model to adapt to more complex power system environments and maintain a high diagnostic accuracy. For the computational cost of the ARO-SVM model, future research could explore an improved version based on lightweight optimization algorithms to reduce the demand for hardware resources. Meanwhile, during deployment, real-time diagnosis through edge computing can reduce latency and improve overall system efficiency. The proposed model is applicable not only to diagnosing cable reverse-connection defects but also to detecting other defects in the HV cable cross-bonded grounding system. In future research, we also need to consider building a more comprehensive and multifunctional diagnostic model. During the actual operation process, the operators need to pay special attention to the following points: Firstly, before the cable line is put into operation, the correct installation and calibration of the current sensors must be ensured. During the cable line operation, the sensors and data acquisition modules should be regularly checked to ensure real-time data updates and validity. To effectively reduce cable reverse-connection defects, operators should also conduct comprehensive connectivity checks before cable installation.

In conclusion, the application of the cable reverse-connection defect diagnosis model based on ARO-SVM in the HV cable grounding system has high potential and can effectively identify reverse-connection defects and improve the reliability of the power system.

## 6. Conclusions

This paper proposed a method to judge cable reverse-connection defects by using the sheath current amplitude in the grounding boxes at both ends of the HV cable. An equivalent model of the HV cable cross-bonded grounding system was established, and defect diagnosis was carried out with the help of simulation and ARO-SVM models. The conclusions were as follows:

1. The amplitude of the sheath current will change with different reverse-connection defects. Based on this, different reverse-connection defects can be diagnosed.
2. The ARO algorithm was used to optimize the SVM model. The diagnostic accuracy rate of the ARO-SVM model for reverse-connection defects was increased by 6.49%, 1.3%, 9.09%, and 5.84%, respectively, compared with those of the DT, XGBoost, BP, and SVM models. It can be seen from this that the proposed algorithm can effectively diagnose the reverse-connection defects of HV cables.
3. The defects of the HV cable cross-bonded grounding system do not only include the reverse-connection defect. In future research, we will focus on constructing a model with more comprehensive fault identification capabilities.

## Figures and Tables

**Table 1:** Classification of states of HV cable cross-bonded grounding system.

Defect Type	Defect Location	State Number
Normal	/	0
A reversed connection in the cable joint	Misconnection of joint J_A1_	1
	Misconnection of joint J_B1_	2
	Misconnection of joint J_C1_	3
	Misconnection of joint J_A2_	4
	Misconnection of joint J_B2_	5
	Misconnection of joint J_C2_	6
Reversed connections in two cable joints	Misconnection of joint J_A1_ and J_B1_	7
	Misconnection of joint J_B1_ and J_C1_	8
	Misconnection of joint J_C1_ and J_A1_	9
	Misconnection of joint J_A2_ and J_B2_	10
	Misconnection of joint J_B2_ and J_C2_	11
	Misconnection of joint J_C2_ and J_A2_	12
	Misconnection of joint J_A1_ and J_A2_	13
	Misconnection of joint J_B1_ and J_B2_	14
	Misconnection of joint J_C1_ and J_C2_	15
	Misconnection of joint J_A1_ and J_B2_	16
	Misconnection of joint J_B1_ and J_C2_	17
	Misconnection of joint J_C1_ and J_A2_	18
	Misconnection of joint J_B1_ and J_A2_	19
	Misconnection of joint J_C1_ and J_B2_	20
	Misconnection of joint J_A1_ and J_C2_	21

**Table 2:** Simulation parameters of cable [24].

Cable Parameters	Value
Outer diameter of conductor/mm	34.2
Inner diameter of metal sheath/mm	78.6
Outer diameter of metal sheath/mm	82.6
Thickness of semiconductor shielding layer/mm	2
Thickness of insulation layer/mm	16
Outer diameter of cable/mm	94
Relative dielectric constant of XLPE	2.3
Resistivity coefficient of conductor/(nΩ⸱m^−1^)	16.8
Resistivity coefficient of the earth/(Ω⸱m^−1^)	100
Resistivity coefficient of sheath/(nΩ⸱m^−1^)	28.4

**Table 3:** Defect diagnosis accuracy of different models.

State Number	DT	XGBoost	BP	SVM	ARO-DT	ARO-XGBoost	ARO-BP	PSO-SVM	SSA-SVM	ARO-SVM
0	100%	100%	100%	100%	100%	100%	100%	100%	100%	100%
1	100%	100%	100%	100%	88.89%	100%	100%	100%	100%	100%
2	47.62%	100%	0%	77.78%	90.91%	100%	66.67%	100%	83.33%	90.91%
3	100%	100%	0%	0%	100%	100%	62.50%	100%	0%	100%
4	100%	100%	37.50%	37.50%	100%	100%	0%	50%	37.50%	100%
5	100%	100%	100%	100%	100%	100%	100%	100%	100%	100%
6	0%	100%	52.38%	75%	91.67%	100%	100%	100%	100%	100%
7	100%	100%	100%	100%	100%	100%	100%	100%	100%	100%
8	100%	75%	100%	100%	100%	85.71%	100%	100%	100%	100%
9	100%	100%	100%	100%	100%	100%	100%	100%	100%	100%
10	100%	88.89%	100%	100%	100%	100%	100%	100%	100%	100%
11	100%	100%	100%	100%	100%	100%	100%	100%	100%	100%
12	100%	100%	100%	100%	100%	100%	100%	100%	100%	100%
13	100%	100%	100%	100%	100%	100%	100%	100%	100%	100%
14	100%	100%	100%	100%	100%	100%	100%	100%	100%	100%
15	100%	100%	100%	100%	100%	100%	100%	100%	100%	100%
16	100%	100%	100%	100%	80%	100%	100%	100%	100%	100%
17	100%	100%	100%	100%	100%	87.50%	53.85%	100%	100%	100%
18	100%	100%	100%	100%	100%	100%	100%	100%	100%	100%
19	100%	100%	100%	100%	100%	100%	100%	100%	100%	100%
20	100%	100%	100%	100%	100%	100%	100%	100%	100%	100%
21	100%	100%	100%	100%	100%	100%	100%	100%	100%	100%
Accuracy	92.86%	98.05%	90.26%	93.51%	97.40%	98.70%	91.56%	98.05%	95.45%	99.35%

**Table 4:** Robustness test results.

Actual Operating State	Corresponding Number	SNR/dB	Test Results
Normal	0	20	0
		30	0
		40	0
Misconnection of joint J_A1_	1	20	1
		30	1
		40	1
Misconnection of joint J_A1_ and J_B1_	7	20	7
		30	7
		40	7
Misconnection of joint J_A1_ and J_A2_	13	20	13
		30	13
		40	13
Misconnection of joint J_A1_ and J_B2_	16	20	16
		30	16
		40	16
Misconnection of joint J_A1_ and J_C2_	21	20	21
		30	21
		40	21

**Table 5:** Measured values of sheath current [32].

Location	Sheath Current of Phase A/A	Sheath Current of Phase B/A	Sheath Current of Phase C/A
Box No. 9	91.0	7.0	95.2
Box No. 10	6.8	8.3	83.9
Box No. 11	87.0	7.3	4.3
GIS terminal	92.0	96.1	5.0

## Data Availability

The data presented in this study are available upon request from the corresponding author. The data are not publicly available due to privacy restrictions.

## Abbreviations

The following abbreviations are used in this manuscript:

HV	High-Voltage
SVM	Support Vector Machine
ARO	Artificial Rabbits Optimization
